# Comparative evaluation of apical microleakage in bioceramic and epoxy resin-based sealers using dye penetration and SEM analysis: An *in vitro* study

**DOI:** 10.6026/973206300213861

**Published:** 2025-10-31

**Authors:** Abdullah Abdullatif Mahdi, Anshad Zaheer, Nabeela Najeeb A.P, Krishnan Hari, Ranjith K, Rajani E.R

**Affiliations:** 1 Department of Restorative and Dental Implants, Allcare Medical Center, Abu Dhabi, UAE; 2 Department of Conservative Dentistry and Endodontics, Travancore Dental College, Kollam, India; 3 Department of Conservative Dentistry and Endodontics, MES Dental College, Perinthalmanna, Malappuram, Kerala, India; 4 Department of Conservative Dentistry and Endodontics, Mar Baselios Dental College, Kothamangalam, Kerala, India; 5 Department of Public Health Dentistry, KIMS Dental College and Hospital, Amalapuram, Andhra Pradesh, India

**Keywords:** Apical microleakage, bioceramic sealer, epoxy resin-based sealer, dye penetration, SEM analysis, root canal sealers

## Abstract

Root canal therapy success depends on achieving a hermetic apical seal to prevent microbial reinfection. Sealers play a crucial role in minimizing microleakage and ensuring long-term treatment success. In this study of forty extracted teeth, a bioceramic sealer demonstrated significantly less dye penetration (1.42 ± 0.23 mm) than an epoxy resin-based sealer (2.68 ± 0.31 mm, p < 0. 05). Scanning electron microscopy revealed superior interfacial adaptation with fewer gaps in the bioceramic group, while epoxy resin- based sealers showed more voids and detachment. These findings indicate that bioceramic sealers provide improved sealing and adaptation to dentin, enhancing the apical seal quality in endodontic therapy.

## Background:

Root canal therapy aims to eliminate microorganisms from the root canal system and prevent reinfection by creating a hermetic seal [1]. The apical seal is particularly critical as it prevents the percolation of bacteria and their by-products from the periapical tissues into the root canal system [2]. Despite advances in instrumentation and obturation techniques, microleakage remains a significant cause of endodontic failure [3]. Root canal sealers play a vital role in achieving an adequate apical seal by filling irregularities between the gutta-percha and the root canal walls [4]. Various types of sealers have been developed over the years, including zinc oxide-eugenol, glass ionomer, calcium hydroxide, silicone, resin-based, and more recently, bioceramic sealers [5]. Among these, epoxy resin-based sealers have been widely used and considered the gold standard due to their excellent adhesion, dimensional stability, and low solubility [6]. In recent years, bioceramic sealers have gained popularity in endodontics due to their biological properties, including biocompatibility, bioactivity, and antimicrobial activity [7]. These sealers are composed of calcium silicate, calcium phosphate, and other mineral oxides that can form hydroxyapatite upon setting [8]. The formation of hydroxyapatite at the sealer-dentin interface may potentially improve the sealing ability and promote tissue regeneration [9]. Several studies have compared the sealing ability of bioceramic sealers with traditional sealers, with conflicting results [10, 11]. While some studies have reported superior sealing ability with bioceramic sealers [12], others have found no significant difference compared to epoxy resin-based sealers [13]. These discrepancies may be attributed to differences in methodology, sample size, or evaluation techniques. Most previous studies have utilized either dye penetration or fluid filtration methods to evaluate microleakage [14]. However, each method has its limitations, and combining multiple evaluation techniques may provide a more comprehensive assessment of the sealing ability [15]. Scanning electron microscopy (SEM) analysis allows for detailed examination of the sealer-dentin interface, which can complement the findings from dye penetration studies [16]. Despite the growing popularity of bioceramic sealers, there is limited research comparing their apical sealing ability with epoxy resin-based sealers using both dye penetration and SEM analysis [17]. Furthermore, the long-term stability of the sealer-dentin interface under simulated physiological conditions requires further investigation [18]. Therefore, this study aimed to compare the apical sealing ability of a bioceramic sealer with that of an epoxy resin-based sealer using both dye penetration and SEM analysis. The null hypothesis was that there would be no significant difference in apical microleakage between the two sealers.

## Materials and Methods:

## Study design:

This was an *in vitro* comparative study conducted at the Department of Conservative Dentistry and Endodontics.

## Sample selection:

A total of 40 extracted single-rooted human teeth were collected from the Oral and Maxillofacial Surgery Department. Teeth were extracted for periodontal or orthodontic reasons and stored in 0.1% thymol solution at 4°C until use. The sample size was determined based on a power analysis with α = 0.05, β = 0.2, and effect size = 1.2, indicating that 20 samples per group would be sufficient to detect significant differences.

## Inclusion criteria:

[1] Single-rooted teeth with fully formed apices

[2] Straight root canals (curvature less than 10°)

[3] No visible cracks or fractures on external surface

[4] No previous endodontic treatment

[5] Tooth length of 19-21 mm

## Exclusion criteria:

[1] Teeth with root caries

[2] Teeth with calcified canals

[3] Teeth with internal or external resorption

[4] Teeth with immature apices

[5] Teeth with anatomical abnormalities

## Materials used:

[1] Bioceramic sealer: EndoSequence BC Sealer (Brasseler USA, Savannah, GA, USA)

[2] Epoxy resin-based sealer: AH Plus (Dentsply Sirona, York, PA, USA)

[3] Gutta-percha points: ProTaper Universal (Dentsply Sirona, York, PA, USA)

[4] Rotary instruments: ProTaper Universal (Dentsply Sirona, York, PA, USA)

[5] Irrigants: 5.25% sodium hypochlorite, 17% EDTA, distilled water

[6] Dye: 2% methylene blue solution (Sigma-Aldrich, St. Louis, MO, USA)

[7] Epoxide resin: EpoFix resin (Struers, Ballerup, Denmark)

## Tooth preparation:

All teeth were decoronated at the cementoenamel junction using a diamond disc under water cooling to obtain a standardized root length of 16 mm. A size 10 K-file (Dentsply Sirona) was inserted into each canal until it was just visible at the apical foramen. The working length was determined by subtracting 1 mm from this measurement. Canals were instrumented using ProTaper Universal rotary files up to size F3 (30/0.09) according to the manufacturer's instructions. During instrumentation, canals were irrigated with 2 mL of 5.25% sodium hypochlorite after each file change, followed by a final rinse with 5 mL of 17% EDTA for 1 minute and 5 mL of distilled water. Canals were dried with paper points.

## Obturation:

The specimens were randomly divided into two groups (n=20 each) using a computer-generated randomization sequence:

[1] Group I: Obturated with gutta-percha and EndoSequence BC Sealer

[2] Group II: Obturated with gutta-percha and AH Plus sealer

For Group I, the bioceramic sealer was mixed according to the manufacturer's instructions and applied to the canal walls using a lentulo spiral. A master gutta-percha cone (F3) was coated with sealer and inserted to the working length. Lateral condensation was performed using accessory gutta-percha cones (size 20) and a finger spreader until the entire canal was filled. For Group II, the epoxy resin-based sealer was mixed according to the manufacturer's instructions (base and catalyst paste in 1:1 ratio). The same obturation technique as described for Group I was used. After obturation, the coronal access was sealed with Cavit G (3M ESPE, Seefeld, Germany), and specimens were stored at 37°C and 100% humidity for 7 days to allow complete setting of the sealers.

## Dye penetration test:

All specimens were coated with two layers of nail varnish, except for the apical 2 mm. The teeth were immersed in 2% methylene blue solution at 37°C for 72 hours. After removal from the dye, the specimens were rinsed under running water for 10 minutes and dried. The nail varnish was removed using a scalpel, and the roots were split longitudinally in a buccolingual direction using a diamond disc and a chisel. The maximum linear dye penetration was measured by two independent examiners using a digital caliper (Mitutoyo, Kawasaki, Japan) under a stereomicroscope at 20x magnification. The measurements were taken from the apical terminus to the most coronal extent of dye penetration along the canal walls. In case of disagreement between the examiners, a third examiner was consulted, and the mean value was recorded.

## SEM analysis:

Five randomly selected specimens from each group were prepared for SEM analysis. These specimens were sectioned transversely at 3 mm and 6 mm from the apex using a low-speed diamond saw under water cooling. The sections were polished with increasingly fine abrasive papers (600, 800, and 1200 grit) and cleaned ultrasonically in distilled water for 5 minutes. The samples were then dehydrated in a graded ethanol series (50%, 70%, 90%, and 100%) for 15 minutes each and allowed to air dry.

The specimens were mounted on aluminum stubs, sputter-coated with gold, and examined under a scanning electron microscope (JEOL JSM-IT500, Tokyo, Japan) at 15 kV. Images were captured at 1000x magnification to evaluate the sealer-dentin interface. The presence of gaps, voids, and adaptation of the sealer to the dentinal walls was assessed by two independent examiners using a scoring system:

[1] Score 0: No gaps, perfect adaptation

[2] Score 1: Minimal gaps (< 5 µm)

[3] Score 2: Moderate gaps (5-10 µm)

[4] Score 3: Severe gaps (> 10 µm) or sealer detachment

## Statistical analysis:

Data were analyzed using SPSS software version 25.0 (IBM Corp., Armonk, NY, USA). The normality of data distribution was assessed using the Shapiro-Wilk test. For dye penetration measurements, the independent t-test was used to compare the two groups. For SEM analysis, the Mann-Whitney U test was used to compare the scores between groups. Inter-examiner reliability was assessed using the intraclass correlation coefficient (ICC) for dye penetration measurements and Cohen's kappa for SEM scores. A p-value of less than 0.05 was considered statistically significant.

## Results:

The mean dye penetration values for both groups are presented in Table 1 (see PDF). Group I (bioceramic sealer) showed significantly less mean dye penetration (1.42 ± 0.23 mm) compared to Group II (epoxy resin-based sealer) (2.68 ± 0.31 mm) (p < 0.05). The difference between the groups was statistically significant (t = 14.72, df = 38, p < 0.001). The intraclass correlation coefficient (ICC) between the two examiners was 0.94, indicating excellent agreement. The SEM analysis results are presented in Table 2 (see PDF). Group I (bioceramic sealer) demonstrated superior interfacial adaptation compared to Group II (epoxy resin-based sealer). In Group I, 60% of specimens showed no gaps (Score 0), while the remaining 40% had minimal gaps (Score 1). In contrast, Group II showed no specimens with perfect adaptation, with 20% having minimal gaps (Score 1), 60% having moderate gaps (Score 2), and 20% having severe gaps or sealer detachment (Score 3). The difference between the groups was statistically significant (U = 3.5, p = 0.004). The Cohen's kappa value for inter-examiner agreement was 0.87, indicating excellent agreement. The overall comparison between the two groups is presented in Table 3 (see PDF). The bioceramic sealer group showed significantly better performance in both dye penetration and SEM analysis compared to the epoxy resin-based sealer group.

## Discussion:

The results of this study demonstrated that the bioceramic sealer (EndoSequence BC Sealer) exhibited significantly less apical microleakage compared to the epoxy resin-based sealer (AH Plus) when evaluated using dye penetration and SEM analysis. These findings are consistent with several recent studies that have reported superior sealing ability of bioceramic sealers [19, 20]. The superior sealing ability of bioceramic sealers can be attributed to their unique properties. Bioceramic sealers have the ability to form hydroxyapatite upon contact with tissue fluids, which can chemically bond to the dentinal walls and create a more intimate seal [21]. This bioactive property was evident in the SEM analysis, which showed excellent adaptation of the bioceramic sealer to the dentinal walls with minimal gaps. In contrast, the epoxy resin-based sealer demonstrated more frequent interfacial voids and areas of detachment, which may have contributed to the higher dye penetration values. The mean dye penetration for the bioceramic sealer group (1.42 ± 0.23 mm) was comparable to values reported in previous studies [22, 23]. However, the dye penetration for the epoxy resin-based sealer group (2.68 ± 0.31 mm) was slightly higher than some previous reports [24, 25]. This discrepancy may be attributed to differences in methodology, such as the type of dye used, immersion time, and storage conditions. The SEM analysis provided valuable insights into the sealer-dentin interface. Additionally, the alkaline pH of bioceramic sealers may contribute to their antimicrobial properties and ability to neutralize bacterial byproducts, further enhancing their sealing ability [26, 27]. The results of this study have important clinical implications. The superior sealing ability of bioceramic sealers may contribute to improved long-term outcomes of root canal treatment by reducing the risk of reinfection. This is particularly relevant in cases with complex anatomy or where complete disinfection of the root canal system is challenging [28]. Furthermore, the biocompatibility and bioactivity of bioceramic sealers may promote periapical healing and tissue regeneration [29]. However, it is important to acknowledge the limitations of this *in vitro* study. The extracted teeth used in this study may not accurately represent the clinical situation, where factors such as pulp vitality, periapical pathology, and occlusal forces may influence the performance of sealers [30]. Additionally, the short-term storage period (7 days) may not reflect the long-term stability of the sealer-dentin interface. Future studies should evaluate the sealing ability of these sealers over longer periods and under conditions that more closely simulate the clinical environment. Another limitation is the use of dye penetration as a method for evaluating microleakage. While this method is widely used and relatively simple, it has been criticized for its inability to detect very small gaps and the potential for the dye to dissolve the sealer [31]. The combination of dye penetration with SEM analysis in this study helped to overcome some of these limitations by providing both quantitative and qualitative assessment of the sealing ability. The findings of this study suggest that bioceramic sealers may offer an advantage over epoxy resin-based sealers in terms of apical sealing ability. However, clinical success depends on multiple factors, including proper instrumentation, irrigation, and obturation technique, in addition to the choice of sealer [32]. Further research is needed to evaluate the long-term clinical performance of bioceramic sealers and their impact on treatment outcomes.

## Conclusion:

Within the limitations of this *in vitro* study, the bioceramic sealer (EndoSequence BC Sealer) demonstrated superior apical sealing ability compared to the epoxy resin-based sealer (AH Plus). The bioceramic sealer showed significantly less dye penetration and better adaptation to the dentinal walls as observed under SEM. Thus, we show that bioceramic sealers may offer an advantage in improving the long-term success of endodontic treatment by providing a more effective apical seal. However, further clinical studies are needed to validate these results and evaluate the long-term performance of these materials *in vivo*.
